# Early markers of right heart involvement in regular smokers by Pocket Size Imaging Device

**DOI:** 10.1186/s12947-015-0024-5

**Published:** 2015-07-23

**Authors:** Vincenzo Schiano-Lomoriello, Roberta Esposito, Ciro Santoro, Giovanni de Simone, Maurizio Galderisi

**Affiliations:** Hypertension Research Center (CIRIAPA), Federico II University Hospital, Naples, Italy; Department of Translational Medical Sciences, Federico II University Hospital, Naples, Italy; Department of Advanced Biomedical Sciences, Federico II University Hospital, Via S. Pansini 5,bld 1, Naples, 80131 Italy

**Keywords:** Smoke, Cardiac ultrasound, Pocket size imaging device, Inferior vena cava, Right atrial diameter

## Abstract

**Purpose:**

To test the diagnostic power of Pocket Size Imaging Device (PSID) in detecting early signs of right heart (RH) involvement in regular smokers (RS) free of overt cardiac involvement.

**Methods:**

One-hundred-forty-three regular smokers and 51 healthy controls, comparable for age and sex, underwent physical exam (PE), PSID exam and standard echocardiography. Based on a simplified Boston score, ≥1 of clinical signs (jugular venous distension, hepatomegaly, peripheral pitting oedema and abnormal pulmonary sounds) were considered indicative of RH involvement. A composite score (1 to 4) obtained by summing the points of four quantitative RH abnormalities detectable by PSID (inferior vena cava [IVC] dilatation, reduced IVC respiratory variation, right ventricular dilatation and right atrial dilatation), was generated and ≥1 of PSID abnormal signs was considered indicative of RH involvement.

**Results:**

Boston score was not significantly different between the two groups. By using PSID, smokers exhibited greater IVC diameter (p < 0.0001), right atrial diameter (p < 0.002) and higher PSID score (p < 0.005) than controls. Compared to PE, the additional diagnostic power of PSID (≥1 abnormal sign of both Boston and PSID score) was 44.9 % in smokers. By dividing smokers in tertiles according to number of cigarettes per day, the third tertile showed the largest values of both IVC and right atrial dimension. Differences were confirmed by standard echocardiography. Reproducibility of PSID measurements and concordance of linear measurements between PSID and standard echo measurements was very good except for concordance of right ventricular basal diameter.

**Conclusions:**

PSID detects early ultrasound signs of RH involvement in regular otherwise healthy smokers in comparison with PE.

## Introduction

An estimated 20 % of american adults are regular smokers [1], without substantial prevalence changes between 1998 and 2007 [2]. About 443,000 people in United States and 695.000 in Europe die annually from “smoking-related” illnesses [3, 4]. Cigarette smoking is a main determinant of chronic obstructive pulmonary disease (COPD), which leads to pulmonary arterial hypertension (PAH) in about 50 % of adults with advanced COPD [5]. The sequence of changes leading to PAH in current smokers begins at early stages with endothelial dysfunction, induced by hypoxia, and inflammation [6]. Right ventricular (RV) failure is a direct consequence of PAH. The specific mechanisms underlying the development of RV failure secondary to PAH are unclear. RV afterload mismatch, myocardial ischemia, microvascular dysfunction, and myocytes apoptosis might be involved [7]. Given the difficulty of treating heart failure with PAH, a primary need correspond to an early identification of patients at risk of PAH and right heart (RH) involvement. To date, early markers of RH involvement can be detected in preclinical stages by using standard echocardiography and normal echocardiographic reference values of right chambers size and of RV function are recognized [8].

Pocket Size Imaging Device (PSID), an ultrasound machine which cannot be classified as a standard echocardiographic machine because of the impossibility of calculating cardiac volumes and quantifying valvular flow by pulsed or continuous wave Doppler, has a potential value as a screening tool [9], because of its extreme manageability and possible use in multiple clinical settings. PSID has already shown good feasibility and accuracy in detecting markers of RH involvement [10]. Accordingly, the present study was designed to assess whether PSID may be used to detect early ultrasound signs and RH involvement in a population of asymptomatic regular smokers with or without PAH through a validation by standard transthoracic echo-Doppler technology.

## Methods

### Study population

The study population included women and men aged > 40 years, smoking cigarettes since at least 5 years, asymptomatic for dyspnea. The non-smokers control group, recruited among the staff of the Federico II University hospital, included healthy subjects who had never smoked. Obesity (body mass index > 30 Kg/m^2^), arterial systemic hypertension (systolic blood pressure > 140 mm Hg and/or diastolic blood pressure > 90 mm Hg or current antihypertensive treatment) and dyslipidemia (total cholesterol > 200 mg/dL and or triglycerides > 150 mg/dl) were not exclusion criteria in both the groups. Patients with coronary heart disease, primary cardiomyopathies, more than mild heart valve disease, atrial fibrillation, congenital heart disease, autoimmune disease, diabetes mellitus and any kind of renal and/or hepatic diseases as well as patients taking beta-blocker drugs and/or diuretics were excluded. The study protocol was approved by the Institutional Ethical Committee of Federico II University Hospital and written informed consent was obtained from all participants.

### Procedures

In the same day (morning time between 9 and 11 am) all the participants underwent sequentially the following diagnostic steps:

1. A clinical history and complete physical exam (including measurement of body weight and height, blood pressure and heart rate) performed in our outpatient clinic by a clinician (CS);
2. An ultrasound assessment by PSID (Vscan, GE, Horten, Norway), performed in the same outpatient clinic by two operators (VS-L or RE), blinded to the physical examination;
3. A complete standard echo-Doppler assessment of the right ventricle, performed in the echo-lab by a cardiologist (MG) blind to both the physical exam and PSID assessment.

Smokers were requested to do not smoke at least two hours before the performance of the clinical exam and cardiac ultrasound (PSID and standard echo) assessment.

The following four clinical signs of RH involvement were considered during physical exam: jugular vein distension, hepatomegaly with hepato-jugular reflux, peripheral pitting oedema, pulmonary crackles. The sum of the points of each sign (0 = absent, 1 = present) was performed to generate a simplified Boston score, as previously described [11].

The following four parameters of right heart involvement were assessed by PSID: 1. maximal (end-expiration) inferior vena cava diameter (IVC) in subcostal view [12, 13], 2. per cent respiratory variation of IVC diameter in subcostal view [12, 13], 3. right atrial (RA) maximal transverse middle diameter in apical 4-chamber view [8], 4. RV maximal transverse basal diameter (RV) in apical 4-chamber view [8]. These four quantitative parameters were used to generate a composite PSID score by summing the points (0 = normal value, 1 = abnormal value) of each parameter. The cut-off values chosen for abnormality of each PSID parameters were the following:

- maximal (end-expiratory) IVC diameter > 2.0 cm [14],
- percent respiratory variation of IVC < 50 % [8, 14],
- RA maximal transverse middle diameter > 4.5 cm [8],
- RV maximal transverse basal diameter > 4.2 cm [14].

By standard transthoracic echo-Doppler exam we measured the same above mentioned four parameters and the following additional parameters, determined according to standardized procedures of our laboratory [15]:

- tricuspid annular plane systolic excursion (TAPSE) [8],
- tricuspid inflow E/A ratio,
- systolic velocity (s’), early diastolic (e’) and atrial (a’) velocities of the tricuspid annulus by pulsed Tissue Doppler,
- retrograde maximal gradient of tricuspid regurgitation by CW Doppler [16], whenever present.

Both PSID and standard echocardiographic exams were performed in left lateral decubitus position.

### Statistical analyses

Statistical analysis was performed by SPSS package release 12 (SPSS Inc, Chicago, IL, USA). Data are presented as mean values ± SD. Inter-group comparison was obtained by one-factor ANOVA and *χ*^2^ distribution with computation of exact p value by Monte Carlo method. The intra-class correlation analysis (rho) was used to test the concordance of the main parameters measured by PSID and standard echocardiographic machine and also intra- and inter-observer reproducibility of PSID measurements. The null hypothesis was rejected at a two-tailed p ≤ 0.05.

## Results

The characteristics of the study population are listed in Table 1. Sex distribution, age, body mass index, blood pressure and heart rate were comparable between the two groups.

**Table 1:** Characteristics of study population

Variable	Controls n = 51	Regular Smokers n = 153	P
Sex (M/F)	24/27	80/71	NS
Age (years)	56.0 ± 7.8	55.5 ± 8.7	NS
BMI (Kg/m^2^)	26.7 ± 4.2	26.7 ± 3.9	NS
Systolic BP (mm Hg)	131.1 ± 14.1	132.5 ± 19.3	NS
Diastolic BP (mm Hg)	79.2 ± 10.1	79.0 ± 10.0	NS
Heart rate (bpm)	70.7 ± 8.9	70.9 ± 10.2	NS
Hypertension (%, n)	50.9 (26)	49.7 (76)	NS
Obesity (%, n)	15.7 (8)	17.0 (26)	NS
Diabetes (%, n)	13.7 (7)	13.1 (20)	NS
Dyslipidemia (%, n)	37.3 (19)	37.9 (58)	NS

BMI = Body mass index, BP = Blood pressure

Table 2 summarizes the data resulting from clinical exam: Boston score was not significantly different between the two groups. The frequence of regular smoker exhibiting at least one clinical sign included in the computation of Boston Score was 11.3 % (n = 6).

**Table 2:** Clinical evaluation

Variable	Controls n = 51	Regular Smokers N = 153	P
Jugular pressure (%)	0 (0)	8 (5.3)	=0.03
Hepatomegaly (%)	1 (1.9)	5 (3.3)	NS
Peripheral oedema (%)	1 (1.9)	4 (2.6)	NS
Pulmonary cracles (%)	1 (1.9)	2 (1.3)	NS
Boston Score	0.06 ± 0.2	0.10 ± 0.3	NS

**Fig. 1:**
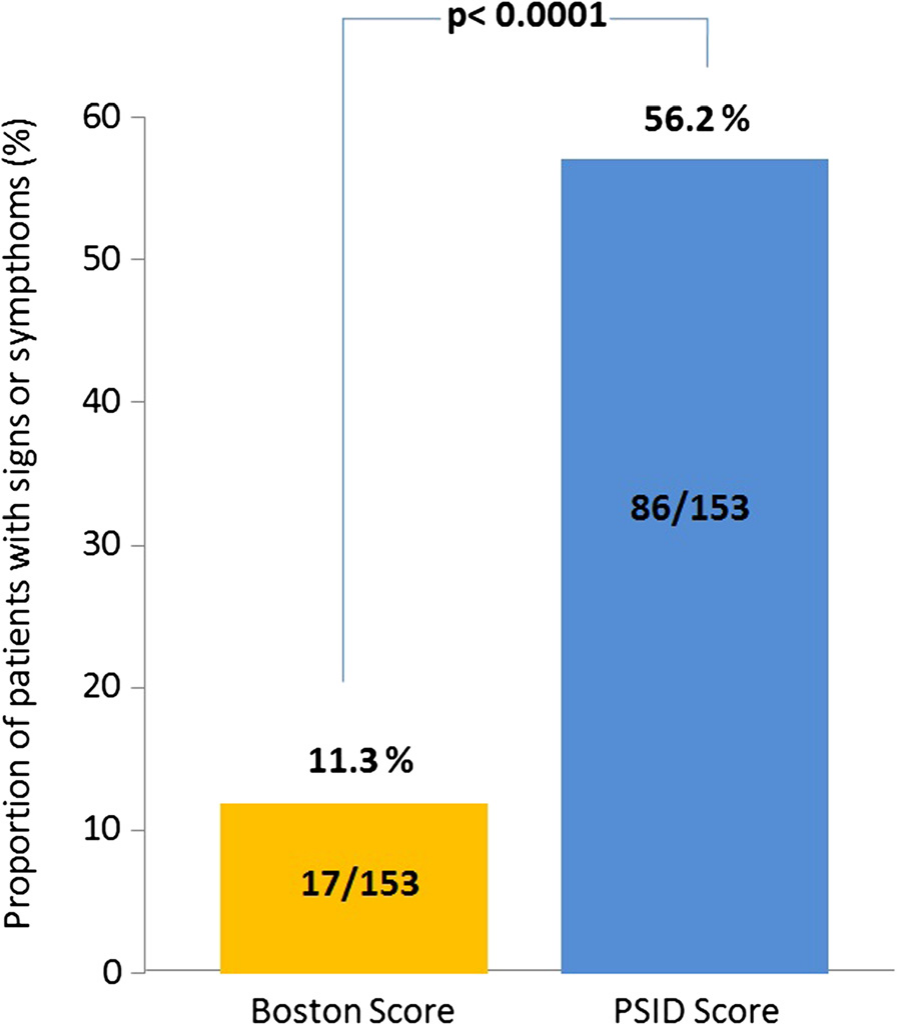
Head to head comparison of the rate of regular smokers with at least one abnormality in simplified Boston score and PSID score. PSID additional diagnostic power appears to be of 44.9 %

Table 3 reports the PSID findings: IVC diameter (p < 0.0001) and RA diameter (p < 0.002) were both higher in regular smokers than in controls. Also PSID score was higher in regular smokers (p < 0.0001). PSID (at least one abnormal ultrasound parameter) extended the detection of RH abnormalities in 86 patients (56 %) in our regular smokers, with an additional diagnostic power of 44.9 % (Fig. 1).

**Table 3:** PSID evaluation

Variable	Controls n = 51	Regular Smokers n = 153	P
IVC diameter (cm)	1.32 ± 0.3	1.59 ± 0.4	<0.0001
Respiratory Variation (%)	51.0 ± 8.3	48.9 ± 9.9	NS
RA diameter (cm)	3.13 ± 0.5	3.39 ± 0.5	<0.002
RV basal diameter (cm)	3.35 ± 0.5	3.49 ± 0.5	NS
PSID Score	0.27 ± 0.5	0.65 ± 0.7	<0.005
IVC dilation (%, n)	2 (1)	8.5(13)	<0.05
Pathological % resp variation (%, n)	5.9 (3)	17 (26)	=0.03
RA dilation (%, n)	0 (0)	3.3 (5)	=0.07
RV dilation (%, n)	3.9 (2)	2.6 (4)	NS

Table 4 reports parameters of right heart obtained by standard echo-Doppler examination: IVC diameter (p < 0.0001) and RA diameter (p < 0.02) were again significantly greater in regular smokers than in controls. Tricuspid regurgitation peak gradient (found in 35 controls and in 128 smokers) and estimated pulmonary arterial systolic pressure did not differ significantly between the two groups.

**Table 4:** Standard Echo Doppler Data of the right ventricle

Variable	Controls n = 51	Regular Smokers n = 153	P
Tricuspid regurgitation gradient (mmHg)*	20.6 ± 5.5	20.6 ± 6.4	NS
PAPs (mm Hg)*	25.5 ± 5.5	26.6 ± 7.1	NS
IVC diameter (cm)	1.33 ± 0.31	1.59 ± 0.36	<0.0001
Respiratory Variation (%)	51.2 ± 8.42	48.7 ± 10.6	NS
RA diameter (cm)	3.20 ± 0.42	3.43 ± 0.57	<0.02
RV basaldiameter (cm)	2.89 ± 0.45	3.02 ± 0.47	NS
RV longitudinal diameter (cm)	6.09 ± 0.69	6.15 ± 0.77	NS
TAPSE (cm)	23.1 ± 3.45	23.6 ± 3.99	NS
Tricuspid E/A ratio	1.16 ± 0.33	1.15 ± 0.44	NS
Tricuspid annular s’ (cm/s)	0.14 ± 0.3	0.13 ± 0.2	NS
Tricuspid annular e’ (cm/s)	0.12 ± 0.3	0.12 ± 0.3	NS
Tricuspid annular a’ (cm/s)	0.14 ± 035	0.15 ± 0.35	NS

**Fig. 2:**
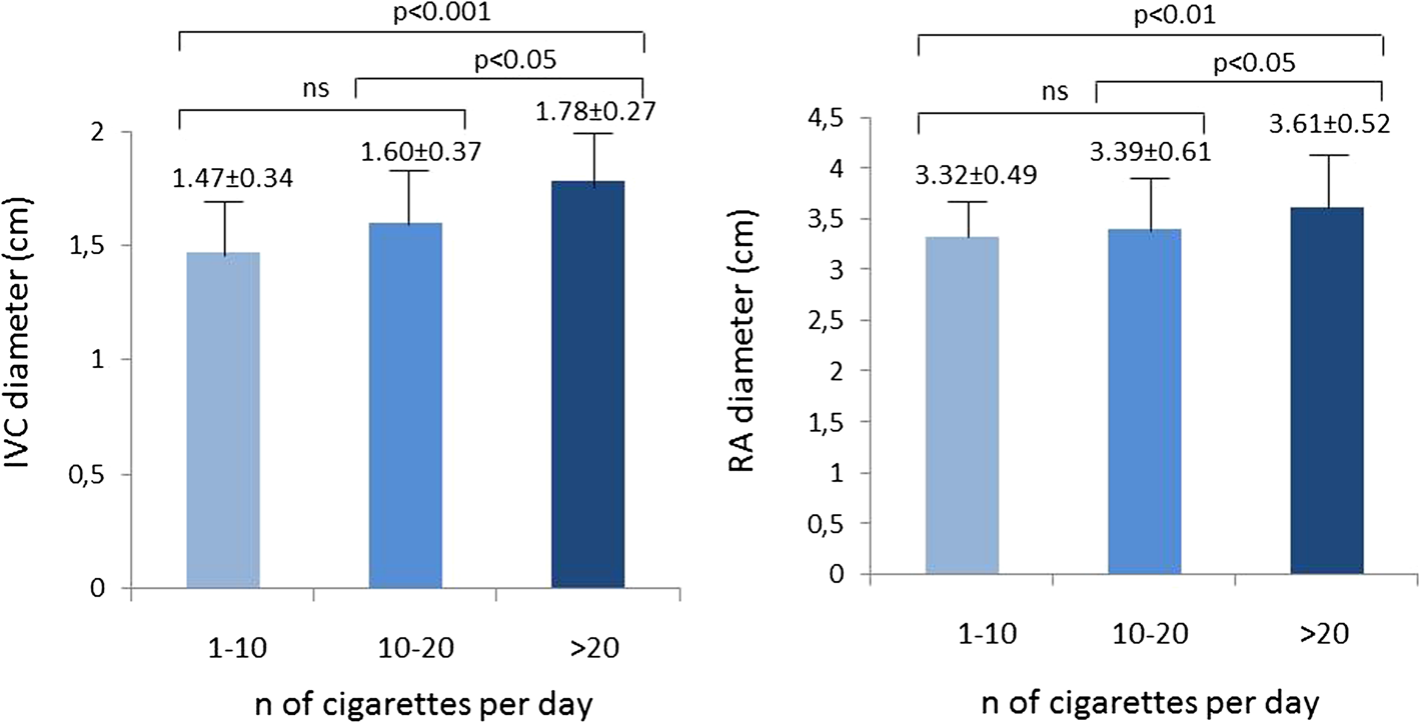
IVC and RA size of regular smokers according to tertiles of cigarettes smoked per day. RA = Right atrial, IVC = Inferior vena cava

Additional analyses were performed by dividing the group of smokers in tertiles according to the number of cigarettes smoked per days (43 smoking 1 to 10 cigarettes/day, 82 smoking 11–20 cigarettes/day and 28 smoking > 20 cigarettes/day). By these analyses both IVC and RA dimension were significantly larger in the third tertile (Fig. 2) whereas IVC respiratory reactivity and RV diameter did not differ significantly among the three tertiles. These results were confirmed also using measures derived from standard echo Doppler exam.

Table 5 shows the intra-class relation coefficients of linear measurements of the right heart between PSID and standard echocardiographic machine: the ρ value was excellent except for RV basal diameter. Also the reproducibility of PSID measurements (data not shown in table) was optimal: rho IVC diameter = 0.96 intra and 0.93 inter-observer, % IVC respitatory variation = 0.97 and 0.96 respectively, RA diameter = 0.98 and 0.96, RV diameter = 0.92 and 0.95).

**Table 5:** Intra-class correlation analysis between PSID and echo standard measurement

Variable	Intra-class Correlation (rho)	95 % Confidence Interval	
		Lower bound	Upper bound
IVC diameter (cm)	0.894	0.860	0.919
IVC percent variation	0.932	0.910	0.949
RA diameter (cm)	0.806	0.745	0.853
RV basal diameter (cm)	0.472	0.304	0.599

## Discussion

PSID is a novel, ultraportable ultrasound machine which provides black and white and colour flow imaging in real-time and allows to calculate only linear measurements and areas of the heart structures. PSID has been already used to complement and extent physical exam [9, 10, 17–20]. Its main cardiac applications include eye-ball quantitation of left ventricular (LV) function [10, 21], detection of LV [9, 19, 21] and RV abnormalities [9], pericardial effusion [9, 21], valve regurgitations [9, 21], aneurysms of abdominal aorta [22] and pleural effusion [23]. PSID could be used also as a screening tool for early detection of cardiac involvement in subclinical stages of heart diseases [9], a potentiality which has been poorly investigated.

The present study is the first to apply PSID to the detection of preclinical markers of RH involvement in asymptomatic regular smokers and reveals that PSID might be useful as a screening tool in this specific clinical setting. We demonstrate that PSID can add useful information to the simple physical exam of these subjects who represent a high risk population for development of PAH and RV failure.

The choice of PSID measurements of right heart we used in the present study has a solid rationale. Maximal diameter and percent respiratory variation of IVC have prognostic value in patients with PAH [12, 24], a recognized complication of smoking habit, with the intermediate condition of COPD [5]. The diameters of the right atrium and right ventricle are also used in the clinical practice as markers of RA and RV dilation respectively [8]. We also generated a composite PSID score determining the total amount of abnormalities for each measurement according to cut-off values of current echo recommendations [8, 14] and comparing this PSID score to the purely clinical simplified Boston score.

Among the examined parameters, IVC respiratory variation and RV diameter did not differ between the two groups, by using both PSID and standard echocardiography. This is likely due to the very early assessment, well before initial symptoms and signs associated with the presence of PAH [12, 25, 26]. However, the evidence of higher IVC size in regular smokers was strengthened by the higher RA diameter. While it is difficult to attribute the increased size of these two parameters to an early increase of RA pressure, it is possible to conceive that nicotine, as a venoconstrictor and sympatethic nervous activator, could have induced central shifting of splanchnic venous blood flow towards IVC and right atrium, causing both to become plethoric. Chronic smokers can have a sustained chronic increase in sympathetic nerve activity due to blunted baroreflexes [27].

The PSID composite score, generated after using recognized clear-cut abnormal values of the four chosen parameters (IVC size and respiratory reactivity, RA and RV diameter) was significantly higher in regular smokers than in controls. In contrast, the simplified Boston score did not recognize any significant difference between the two groups. By comparing Boston and PSID score in smokers (at least 1 abnormal sign/parameter included in both the scores), PSID showed a 44.9 % increase in the capacity of detecting RH involvement in our regular smokers.

The simple physical exam has obvious limitations for diagnosis of heart involvement in subclinical cardiac diseases [28, 29]. A novel clinical approach including the additional support of hand-held echocardiography was already proposed [30–34] and refined combining physical exam and PSID, by a purely visual, clear-cut assessment of main parameters [10]. The present study further extends these findings and the potential indications for the use of PSID.

An additional finding of our study is provided by the analyses we performed dividing the smoker population according to tertiles of daily smoked cigarettes. These analyses was done to highlight possible graduality effect of smoking habit on PSID parameters. Both IVC and RA diameter were larger in the third tertile, i.e. in smokers of more than 20 cigarettes/day. These results, found also by using standard echo machine, highlight further the well known negative effect of cigarette smoking on cardiac and vascular structure [6]. They point out that this effect is proportional to the intensity of nicotine exposure [27].

Globally, the results of the present study are reinforced by the good intra- and inter-observer reproducibility of PSID measurements and by the good intra-class relation coefficients between PSID and standard echo measurements of the right heart. These coefficients were very good for all the quantitative parameters analyzed, with the exception of RV diameter. The difficulty of obtaining a definite border delineation of RV chamber size in apical 4-chamber view by using PSID can explain the weakness of RV diameter using PSID.

### Study limitations

The main limitation of the use of PSID in the explored clinical context was the impossibility of obtaining a non invasive estimation of tricuspid regurgitation gradient and of pulmonary arterial pressure. This contributes to differentiate substantially PSID from a standard echo instrumentation. Accordingly, we *a priori* decided to do not use the simple color assessment of tricuspid regurgitation in our composite PSID score. Although tricuspid regurgitation can be visualized by color Doppler of PSID, the diagnostic accuracy of PSID in distinguishing marginal from clinically significant regurgitation has been found to be poor [10, 20], mainly because of the fix pulse-repetition frequency of PSID which debars an effective graduation of valvular regurgitations.

Another limitation corresponds to the lack of any kind of estimation of RV systolic function and of RA volume by using PSID. Very recent 2015 echo recommendations on chamber quantification encourage the use of RA volume as the most reliable parameter of RA size [35]. However, volumes cannot be estimated by PSID and our study was conceived before the publications of these new recommendations.

The lack of data on lung function tests (e.g. spirometry) and on biomarkers (BNP and/or pro-BNP), not performed because of the screening nature of our asymptomatic smokers, could have been useful to better refine the clinical picture of these subjects and could be considered as a further study limitation.

## Conclusions

We demonstrate that the prompt technology of PSID provides the ability of detecting RH involvement in regular asymptomatic smokers. These data are consistent with an early RV longitudinal dysfunction observed in young, asymptomatic smoker buy using standard echo and strain rate imaging [36]. Changes of both IVC and RA size found in our asymptomatic smokers could be explained on the basis of the venoconstrictor effects of nicotine, able to shift the venous blood flow towards the right venous circulation, thus inducing a IVC overload. PSID might be therefore proposed as an useful screening tool in this clinical setting.
